# Analyses of virus/viroid communities in nectarine trees by next-generation sequencing and insight into viral synergisms implication in host disease symptoms

**DOI:** 10.1038/s41598-019-48714-z

**Published:** 2019-08-22

**Authors:** Yunxiao Xu, Shifang Li, Chengyong Na, Lijuan Yang, Meiguang Lu

**Affiliations:** 1 State Key Laboratory for Biology of Plant Diseases and Insect Pests, Institute of Plant Protection, Chinese Academy of Agricultural Sciences, Beijing, China; 2 Environment and Plant Protection Institute of Chinese Academy of Tropical Agricultural Sciences, Haikou, Hainan, China; 3 The Agricultural Development and Service Center of WFD, Liaoning, China

**Keywords:** High-throughput screening, Virus-host interactions

## Abstract

We analyzed virus and viroid communities in five individual trees of two nectarine cultivars with different disease phenotypes using next-generation sequencing technology. Different viral communities were found in different cultivars and individual trees. A total of eight viruses and one viroid in five families were identified in a single tree. To our knowledge, this is the first report showing that the most-frequently identified viral and viroid species co-infect a single individual peach tree, and is also the first report of peach virus D infecting *Prunus* in China. Combining analyses of genetic variation and sRNA data for co-infecting viruses/viroid in individual trees revealed for the first time that viral synergisms involving a few virus genera in the *Betaflexiviridae*, *Closteroviridae*, *and Luteoviridae* families play a role in determining disease symptoms. Evolutionary analysis of one of the most dominant peach pathogens, peach latent mosaic viroid (PLMVd), shows that the PLMVd sequences recovered from symptomatic and asymptomatic nectarine leaves did not all cluster together, and intra-isolate divergent sequence variants co-infected individual trees. Our study provides insight into the role that mixed viral/viroid communities infecting nectarine play in host symptom development, and will be important in further studies of epidemiological features of host-pathogen interactions.

## Introduction

Peach is one of the most widely grown fruit crops in China, and nectarine (*Prunus persica* cv. *nectarina*) is an important cultivar of peach. Viruses and viroids can cause significant negative effects on fruit quality and yield in peaches and nectarines. Previous reports have shown that apple chlorotic leaf spot virus (ACLSV), plum pox virus (PPV), prunus necrotic ringspot virus (PNRSV), prune dwarf virus (PDV), apple mosaic virus (ApMV), plum bark necrosis stem pitting-associated virus (PBNSPaV), peach latent mosaic viroid (PLMVd), and hop stunt viroid (HSVd) are the major pathogens that infect these trees^1^. Yu *et al*. (2013) performed a large-scale field survey of the major viruses and viroids that infect peach trees in China using RT-PCR and ELISA, and the results showed that only ACLSV, PNRSV, cherry green ring mottle virus (CGRMV), apricot pseudo-chlorotic leaf spot virus (APCLSV), PLMVd, and HSVd were detected^2^. However, next-generation sequencing (NGS) approaches have opened new avenues in recent years for the identification of viruses and viroids (including novel pathogens), and this technology is well suited for large-scale pathogen surveys, not only because it can increase the speed at which a wide range of known pathogens are detected, but also because it can be used to detect newly-emerging or potential pathogens for which other diagnostic tools are not yet available^3–6^. Several NGS-based strategies have been developed to overcome problems with traditional approaches, and these have resulted in the identification of known and novel viruses in peach. These include two novel luteoviruses, nectarine stem-pitting-associated virus (NSPaV)^5,7,8^ and peach-associated luteovirus (PaLV)^9^; two marafiviruses, nectarine virus M (NeVM)^5^ and peach virus D (PeVD)^10^; a novel fabavirus in the family *Secoviridae*, peach leaf pitting-associated virus (PLPaV)^11^, and three very similar members of the genus *Foveavirus*, asian prunus virus 1 (APV1), APV2, and APV3^12^.

In addition, complex mixed infections have been found among fruit tree-infecting viruses^2,5,12,13^. Thus, the potential contribution of each single virus infection to the symptoms observed cannot easily be associated with a disease in the infected *Prunus* trees. In fact, many horticultural plants that are routinely clonally propagated are reservoirs of a large variety of viruses and viroids. The importance of the virome in mammalian biology, and the emerging concept of virome-host interactions and their relationship to host genetics was first described by Virgin (2014)^14^. The virome of the microbiome interactions with the host, especially in mammalian biology, has recently become a hot research topic that relies on bioinformatic tools and NGS technology^14–18^. However, only a limited number of studies have revealed viral communities or viromes in peach^17^. In this study, we used NGS technology to study the viral communities in nectarine trees with different disease phenotypes. We identified both known and novel viruses and viroids, and performed comparative analyses of the potential contribution of the pathogens to disease symptoms. Our results will extend the range and kinds of virus and viroid species that infect peach trees, and provide insight into the viral synergisms and the agents that might be associated with disease symptoms in nectarine.

## Results

### Virus and viroid accumulation and pathogen communities within individual nectarine trees

**Figure 1:**
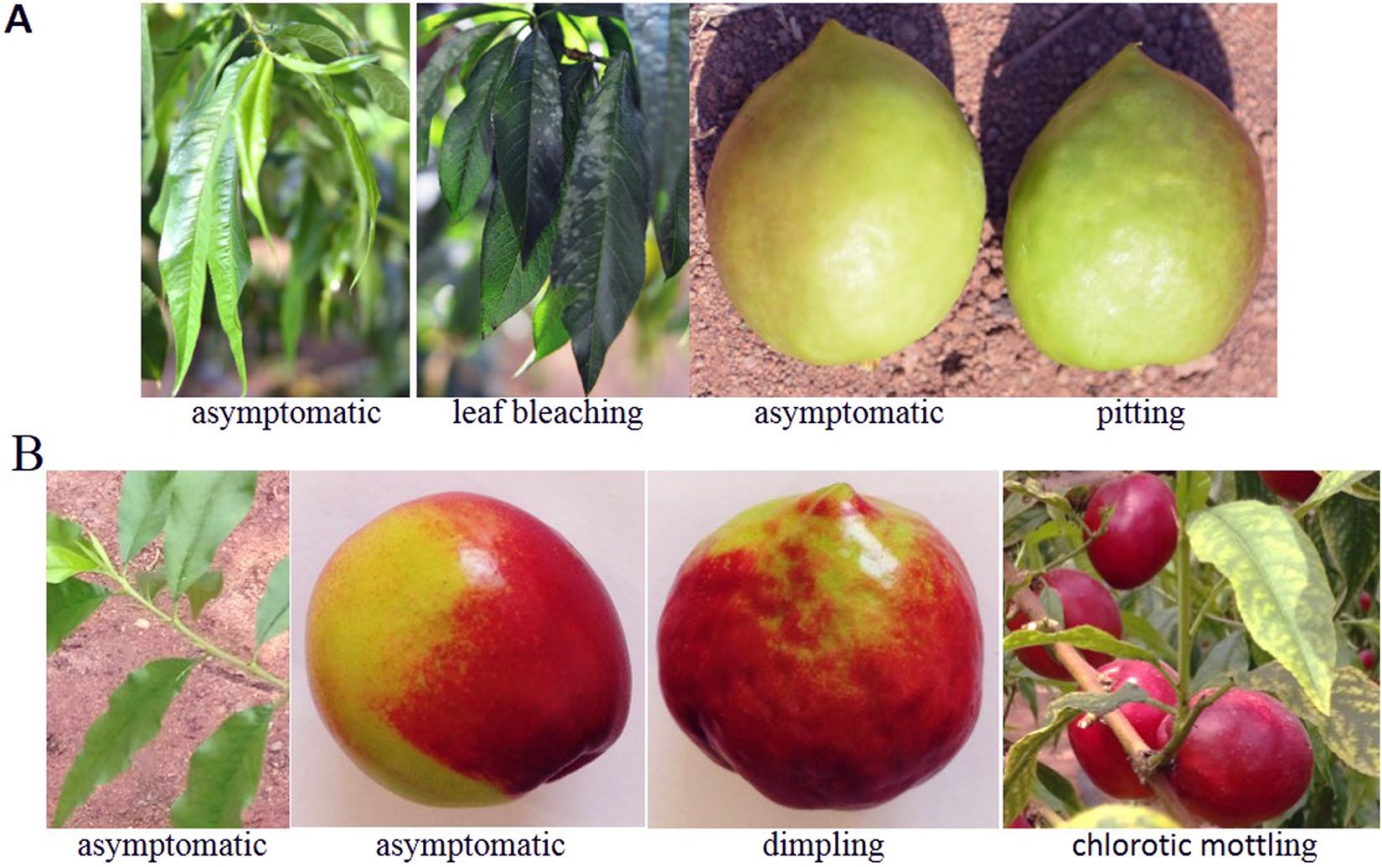
Disease phenotypes of the five nectarine trees sampled for sRNA sequencing. (**A**) Cultivar ‘Youtao 1233’ showing typical symptoms on two trees in greenhouse #1, (**B**) cultivar ‘Zhongyou 4’ showing symptoms on three trees in greenhouse #2.

In the five nectarine tree samples, T01 and T02 were collected in greenhouse #1 from the same nectarine cultivar ‘Youtao 1233’ (10 year-old trees), while T03, T04, and T05 were collected in greenhouse #2 from nectarine cultivar ‘Zhongyou 4’ (5 year-old trees). The five samples came from trees that showed different leaf and fruit symptoms (Table 1, Fig. 1).

**Table 1:** Nectarine tissue samples used for NGS analyses of small RNAs.

Sample	Origin	Cultivar	Leaf and fruit symptoms
T01	Greenhouse 1	Youtao 1233	Leaf bleaching, asymptomatic fruit
T02	Greenhouse 1	Youtao 1233	Leaf bleaching, fruit pitting
T03	Greenhouse 2	Zhongyou 4	Leaf and fruit asymptomatic
T04	Greenhouse 2	Zhongyou 4	Leaves asymptomatic, fruits dimpled
T05	Greenhouse 2	Zhongyou 4	Leaves with chlorotic mottling, asymptomatic fruit

To perform comparative analyses of the different symptoms observed in the nectarine trees, we used NGS of the sRNAs extracted from the five samples to obtain a complete survey of the virus and viroid communities infecting each tree. The Illumina reads obtained from sequencing the five cDNA libraries, which were prepared using RNA extracted from the scion parts of the grafted trees, yielded between 22,434,184 and 30,438,485 raw sRNA reads per library. From these, we obtained between 20,906,239 and 28,435,618 clean reads for samples T01 to T05 (Table S1). A virus and viroid library was constructed from virus and viroid genomes available from NCBI and was then used for mapping of the sRNA reads using the short-sequence alignment program Bowtie. The majority of the reads were 18 to 25 nt in length, with most being either 21 nt or 22 nt. *De novo* assembly of the sRNAs and blastn and blastx searches resulted in assemblies of 15 to 744 contigs, with lengths ranging from 33–474 nt, that were associated with known viruses/viroids. The virus/viroid-associated reads per sample ranged from 1.11% to 9.60% of the clean sRNA reads for the five samples. We found that samples T01 and T02 from greenhouse #1 had the highest number of virus/viroid-associated reads, with 5.53% and 9.60% of the clean sRNA reads, respectively, while the virus/viroid-associated reads in samples T03, T04, and T05 ranged from 1.11% to 2.71% (Table S1).

To compare the different viral communities and the relative numbers of individual viruses and viroids in each sample, we examined individual contig numbers for previously-identified viruses and viroids and calculated the percentage of individual virus or viroid-associated reads by dividing the number of virus or viroid-associated reads by the total number of clean sRNA reads (x 100).

**Figure 2:**
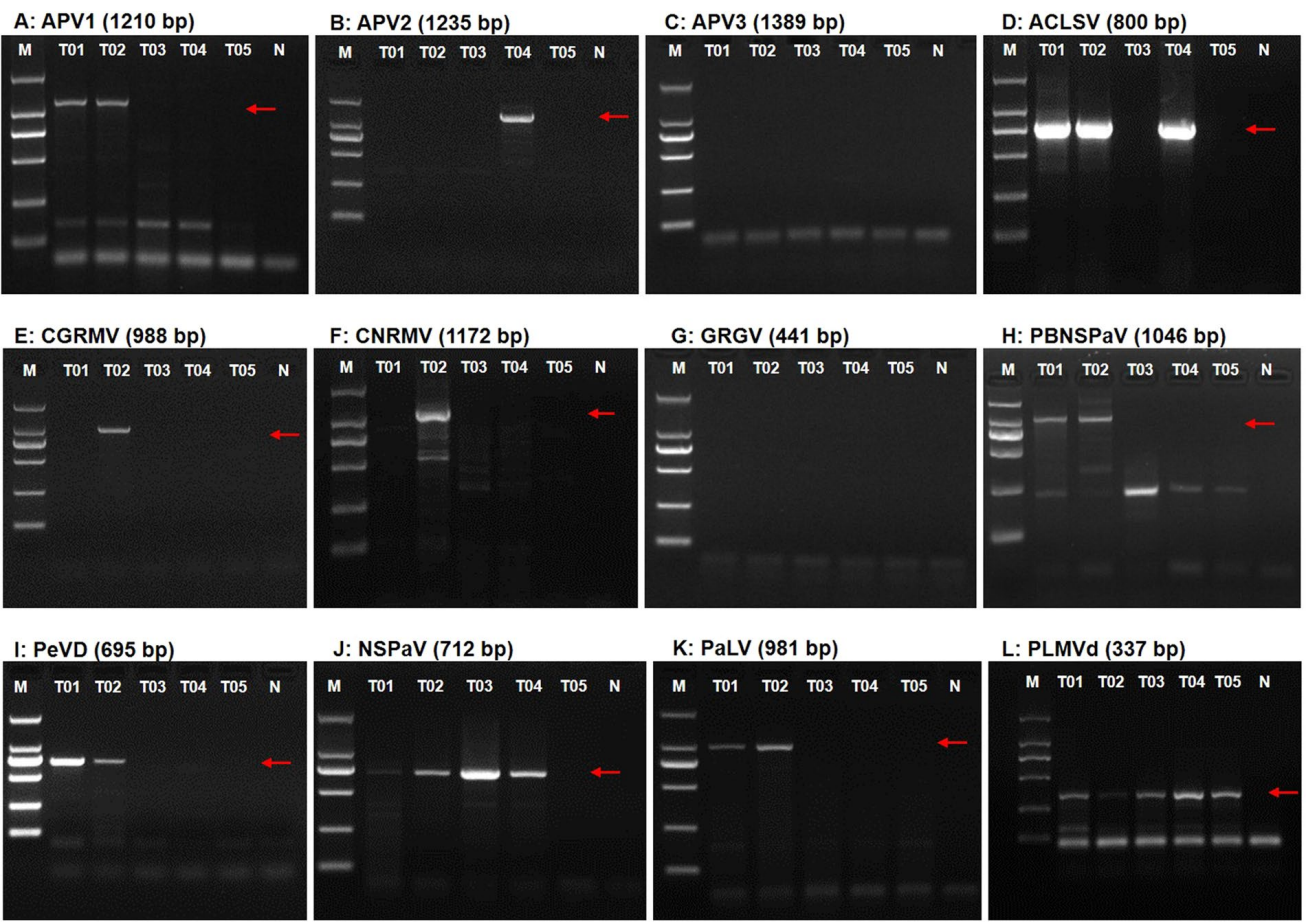
Confirmation of the identified viruses and viroids by RT-PCR. RT-PCR was used to amplify partial sequences of (**A**) APV1, (**B**) APV2, (**C**) APV3, (**D**) ACLSV, (**E**) CGRMV, (**F**) CNRMV, (**G**) GRGV, (**H**) PBNSPaV, (**I**) PeVD, (**J**) NSPaV, (**K**) PaLV, and (**L**) PLMVd using virus or viroid-specific primer pairs from nectarine tree RNA samples T01, T02, T03, T04, and T05. Amplified DNA fragments were examined by electrophoresis on 1.5% agarose gels and stained with EtBr. M, DNA size 2000 marker; N, negative control.

The virus and viroid communities and the numbers of individual pathogens differed between samples T01 and T02 from trees that had different fruit symptoms. Sample T02 had the most identified viral species, and also the highest number of contigs for one viroid and nine virus genera in five families; these included PLMVd in the genus *Pelamoviroid*, (family *Avsunviroidae*); two unassigned viruses NSPaV and PaLV (family *Luteoviridae*); PBNSPaV in the genus *Ampelovirus* (family *Closteroviridae*); ACLSV in the genus *Trichovirus* and APVs in the genus *Foveavirus* (most contigs were identitied that correspond to segments, but a few contigs were identified as APV1 and APV3 that corresponded to segments with high sequence similarities), CGRMV, cherry necrotic rusty mottle virus (CNRMV) in the *Robigovirus* (family *Betaflexiviridae*); PeVD in the genus *Marafivirus* (family *Tymoviridae*); and another 11 contigs that may represent a novel unknown virus in the family *Tymoviridae* which showed identities to segments from *marafi*-, *tymo*-, and *maculaviruses* with low sequence similarities (Fig. 2A). Excluding CGRMV and CNRMV, other seven viruses (PLMVd, NSPaV, PaLV, PBNSPaV, ACLSV, APVs, PeVD) were identified in sample T01. (Fig. 2A). This is also the first report of the identification of PeVD in *Prunus* trees in China.

PBNSPaV and PLMVd (34.32% and 50.45% of the total virus and viroid -associated sequence reads, respectively) were the dominant viruses/viroids identified in sample T01, while these same two pathogens were also the dominant viruses/viroids in sample T02, accounting for 63.75% (PBNSPaV) and 19.74% (PLMV) of the total virus and viroid -associated reads. In addition, CGRMV (5.77% of the total virus-associated reads) and CNRMV (5.77% of the total virus-associated reads) were only detected in sample T02 (Fig. 2B,C). The number of contigs in T01 and T02 that mapped to the PBNSPaV genomes in GenBank was different, and indicated the presence of divergent sequence variants in the two samples (Table 2). We also found that the number of contigs that mapped to several isolates of PBNSPaV (Phm-WH-3, WH-1, PR258-2) collected from plum trees with disease symptoms^19,20^ were significantly increased in sample in T02 (Table 2). By comparing the number of assembled viral contigs and the percentages of virus-associated reads between samples T01 and T02, we found that the T02 sample collected from fruit pitting tree had the higher levels reads of the viruses PBNSPaV, CGRMV, and CNRMV and the higher mapping number of contigs of PBNSPaV isolate from sample with disease symptoms.

**Table 2:** Number of contigs that map to PBNSPaV genomes in GenBank using NGS.

Genome	Host	Symptoms	Origin	Contig numbers	
				T01	T02
KJ792853.1 isolate Plm-WH-3	Plum	Trunk gummosis, cracking, necrosis (19)	China	24	47
KJ792852.1 isolate WH-1	Peach	Trunk gummosis, cracking, necrosis, stem pitting (19)	China	29	49
KC590346.1 isolate PR258-2	GF305 (Peach)	Peach red marbling disease (20)	France	49	52
HG917400.1 isolate VC1	Apricot	Unknown	Italy	6	15
EF546442.1	*Prunus domestica*	Small, chlorotic, distorted leaves^43^	USA	13	18
KC590345.1 isolate Pair-2	GF305 (Peach)	Unknown (20)	France	13	10
KC590344.1 isolate Tatao25 Q-375-02	Peach	Unknown (20)	China	10	6
KJ792854.1 isolate GS-3	Peach	Asymptomatic (19)	China	13	9

**Figure 3:**
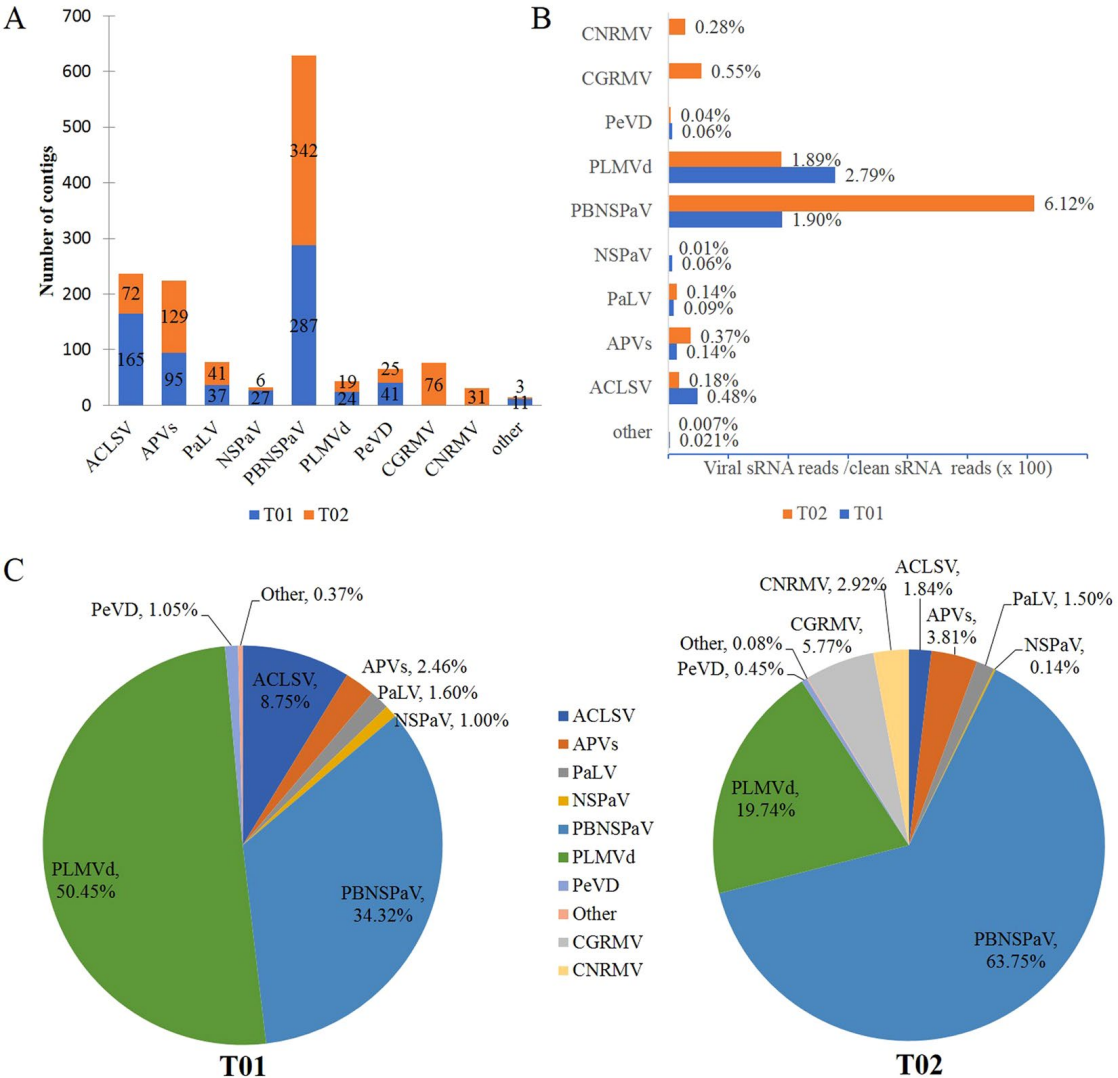
Identification and quantification of the pathogen-associated sequencing reads for the viruses/viroids infecting tree samples T01 and T02 using sRNA sequencing. (**A**) The numbers of assembled viral contigs associated with known viruses and viroids in samples T01 and T02 from greenhouse #1. (**B**) The percentage of virus or viroid-associated reads in each sample was calculated as the number of viral reads divided by the number of clean sRNA sequencing reads (x 100). (**C**) And by the total number of pathogen reads from all identified viruses and viroids (x 100). The reads from APV1 and APV3 were combined (“APVs”) due to their high degree of sequence similarity. Sequencing reads that aligned to another 11 contigs with identities to marafi-, tymo-, and maculaviruses, corresponding to segments with low sequence similarity that may be a novel unknown virus in the family *Tymoviridae*, were combined as “other”.

The viral communities and the numbers of the individual virus varied significantly in the three samples with diverse disease phenotypes (T03, T04 and T05). In the asymptomatic sample T03, we only identified contigs associated with two known viruses/viroids (PLMVd and NSPaV), while in sample T04, which had dimpled fruits, we identified contigs from another two viruses (ACLSV and APV2) in addition to PLMVd and NSPaV (Fig. 3A). Figure 3(B,C) shows that PLMVd and NSPaV accounted for the highest percentages of the individual pathogen-associated reads and were the dominant pathogens in samples T03 and T04. From these results, we can infer that higher levels of ACLSV and APV2 co-infection or their interactions with PLMVd or NSPaV may be associated with the fruit dimpling symptoms seen in sample T04. Sample T05 with chlorotic mottle leaf symptoms contained mainly PLMVd sequences (14 assembled contigs, 2.27% of the total clean reads), and one contig that showed identity to sequences related to a segment (68/84 nt) of GRGV in the genus *Maculavirus*, (family *Tymoviridae*), but the read number was very low (0.0012% of the total clean reads) and this will need to be further confirmed by subsequent RT-PCR to determine whether the low level of reads observed resulted from sample contamination (Fig. 3).

### Confirmation of identified viruses and viroids by RT-PCR

**Figure 4:**
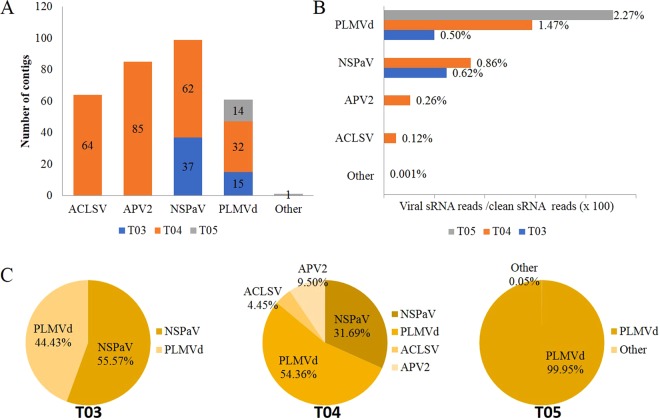
Identification and quantification of the pathogen -associated sequencing reads for the viruses/viroids infecting tree samples T03, T04, and T05 using sRNA sequencing. (**A**) The numbers of assembled viral contigs associated with known viruses and viroids in samples T03, T04 and T05 from greenhouse #2. The percentage of virus and PLMVd-associated reads (**B**,**C**) in each sample was calculated as described in Fig. 3 for samples T03, T04, and T05.

In order to determine whether the viruses and viroids identified by NGS were actually present in the five nectarine tree samples, we conducted RT-PCR using the primer pairs specific for the individual viruses/viroids (Table S2). We found that it was very difficult to distinguish between very closely related viruses such as APV1, APV2, and APV3, and CGRMV and CNRMV, and to identify the presence of viruses that had very few and short contigs with low read numbers in the NGS data. The RT-PCR results obtained, followed by Sanger sequencing, indicated that excluding APV3 and grapevine red globe virus (GRGV), the samples were positive for all other viruses identified by NGS, suggesting that these viruses were actually present in the sRNA extracted from the nectarine tree samples (Fig. 4). In order to further confirm the results of the NGS screen, we also detected individual viruses/viroids in 36 samples collected from different greenhouses and cultivars using RT-PCR assays, and these results are summarized in Table S4. RT-PCR detection indicated that the other test samples (T01-type: N8, N8-2; T02-type: N9, N9-2; T05-type: P12, P13, P14; T03-type: P25, P26, P27; T04-type: P20, P21, P23, P24) with disease symptoms similar to those from the five individual trees (T01, T02 and T03, T04, and T05), respectively, had uniform virus and viroid communities that agreed with the NGS data. The different mixed virus/viroid communities which were detected in the different cultivars were also confirmed.

### Phylogenetic analysis of the identified viruses/viroids

**Figure 5:**
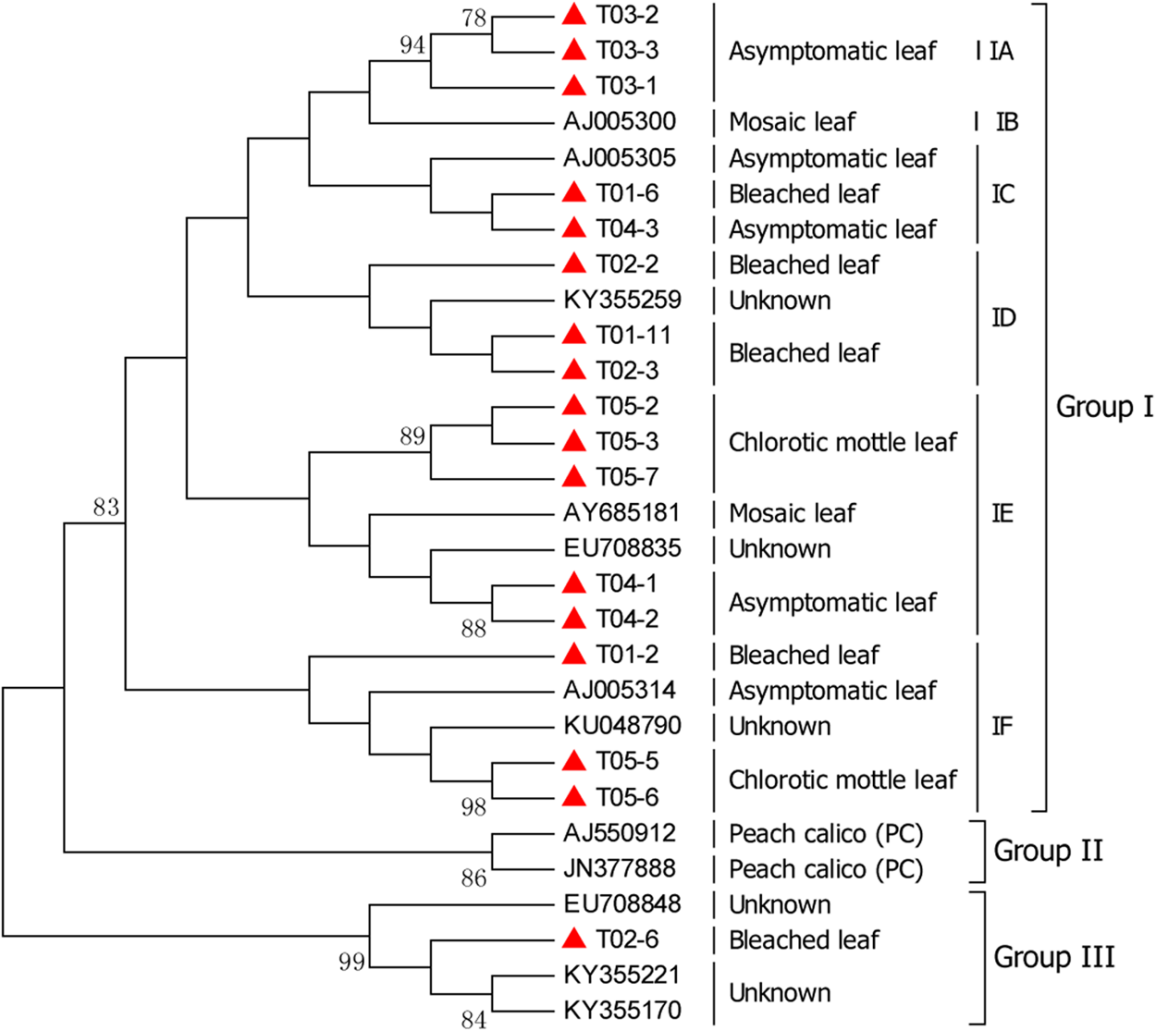
Phylogenetic analysis of PLMVd isolates. The phylogenetic tree was constructed by the maximum-likelihood (ML) method using MEGA6 software. Bootstrap confidence values (1,000 replicates) are given at the branch nodes. Branches corresponding to partitions reproduced in <75% of bootstrap replicates are collapsed. The PLMVd genome sequences obtained in this study are marked with a red triangle.

We identified PLMVd infection in all five tested nectarine trees, and 17 complete genome sequences of PLMVd isolates were obtained from the five samples by RT-PCR and cloning. A combined phylogenetic analysis of the genomic sequences of the PLMVd isolates from this study with some PLMVd sequences in GenBank gave three major phylogroups, and we found that the PLMVd sequences associated with symptomatic and asymptomatic trees did not all cluster together, except for two previously-reported peach calico (PC) isolates^21^ (which clustered alone in Group II; Fig. 5). Further pairwise comparisons of the 17 PLMVd genome sequences showed that the PLMVd isolates from the nectarine samples are quite divergent, and share between 80.0 and 99.7% nucleotide identity. Of the five sampled trees, three sequences from the T03 isolate (asymptomatic sample) that clustered in subgroup IA shared a high degree of nucleotide identity (98.5–98.8%), whereas sequences from the other four sampled trees were highly variable, and clustered into different subgroups, IA to IF (Fig. 5). These results suggest that intra-isolate, genetically distinct sequence variants in individual nectarine trees were present in four of the tree samples (T01, T02, T04, and T05) based on the distribution of sequences in different phylogroups. We conclude that divergent sequence variants that co-infect individual trees is common.

**Figure 6:**
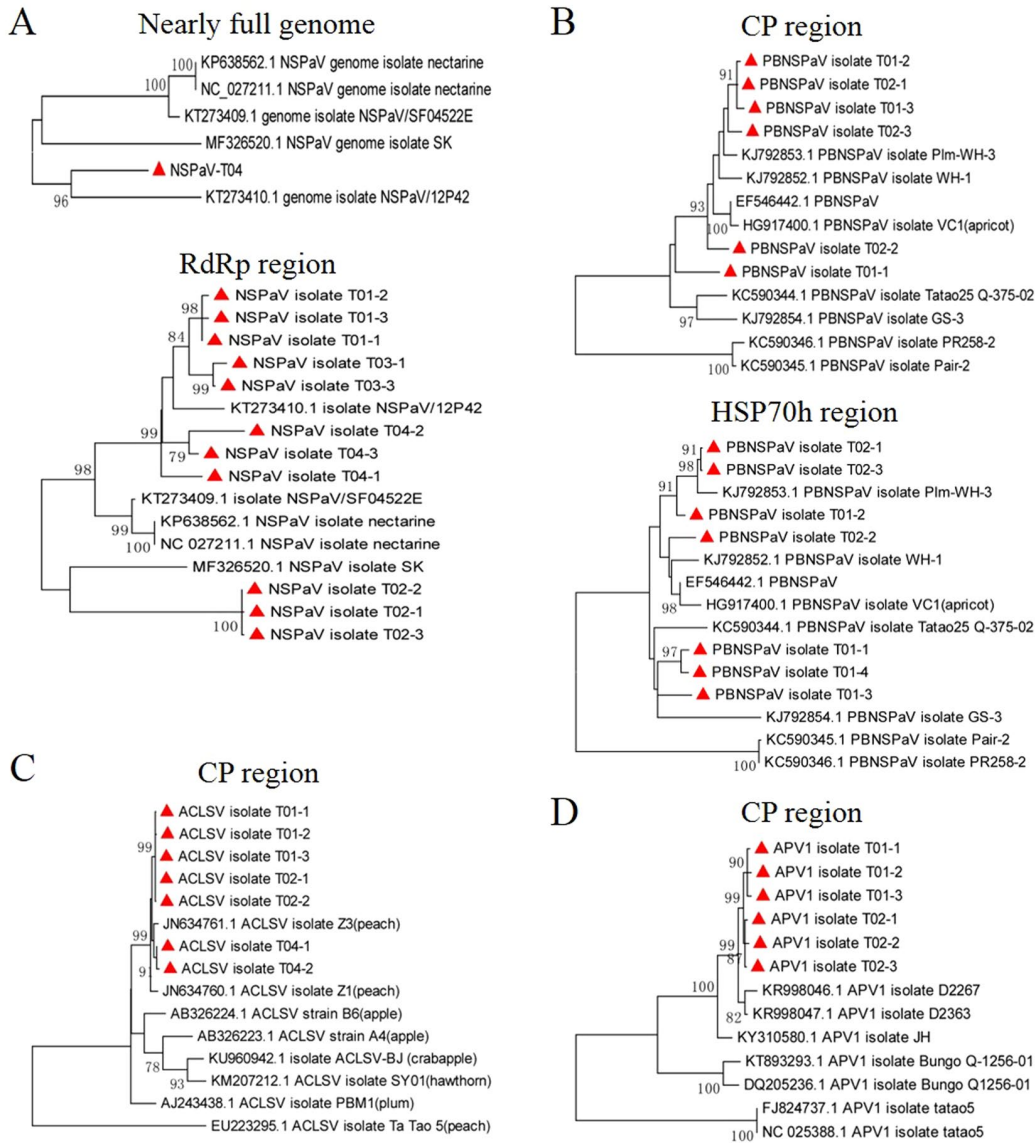
Phylogenetic analysis of the identified viruses. The phylogenetic trees were constructed as for PLMVd in Fig. 5. The viral gene sequences obtained in this study are marked with a red triangle. (**A**) The nearly full genome and RdRp gene of NSPaV; (**B**) the CP and HSP70h genes of PBNSPaV; (**C**) the CP gene of ACLSV; (**D**) the CP gene of APV1.

**Figure 7:**
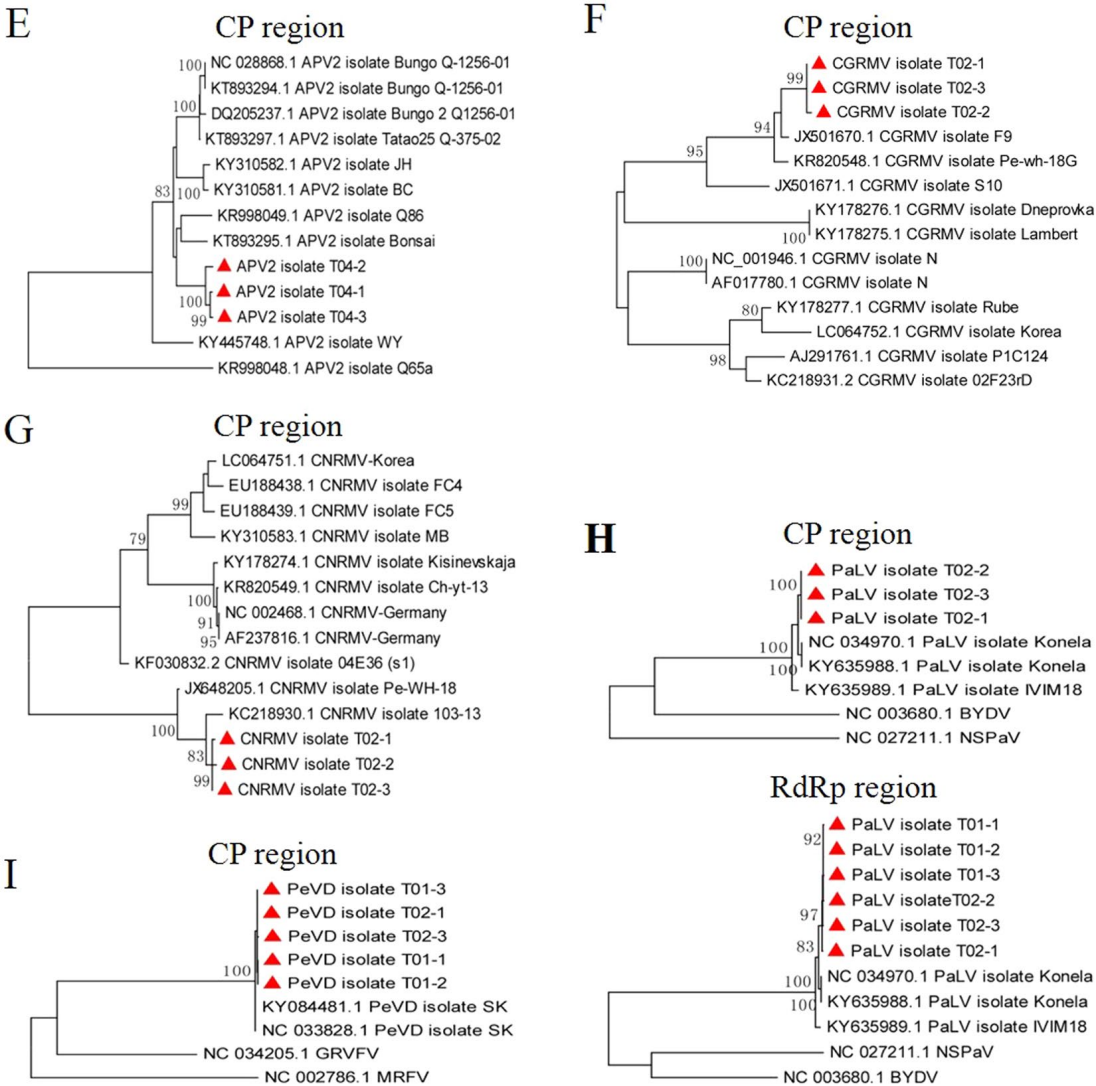
Phylogenetic analysis of the identified viruses. The phylogenetic trees were constructed as for PLMVd in Fig. 5. The viral gene sequences obtained in this study are marked with a red triangle. (**E**) The CP gene of APV2; (**F**) the CP gene of CGRMV; (**G**) the CP gene of CNRMV; (**H**) the CP and RdRp genes of PaLV; (**I**) the CP gene of PeVD. NSPaV and barley yellow dwarf virus (BYDV) were used as outgroups in the PaLV tree. Grapevine rupestris vein feathering virus (GRVFV) and maize rayado fino marafivirus (MRFV) were used as outgroups in the PeVD tree.

In the same way, we constructed two phylogenetic trees based on one nearly complete genome sequence (excluding the 5’ and 3’ terminal sequences) and 11 RNA-dependent RNA polymerase (RdRp) gene sequences from NSPaV isolates T01, T02, T03, and T04 (Fig. 6A). Three NSPaV RdRp gene sequences from T02 grouped with isolate SK from a nectarine in South Korea^22^. Other sequences including RdRp and nearly complete genome sequences of the T01, T03, T04 NSPaV isolates were found to be closely related to isolate NSPaV/12P42 derived from a nectarine in the USA^5^, but three NSPaV RdRp gene sequences from T04 were not consistant, and the T04-1 sequence was detected as a recombinant. Six coat protein (CP) gene sequences from PBNSPaV isolates from T01 and T02 are closely related to the known isolates Plm-WH-3 and WH-1^19^ from peach in China. One PBNSPaV CP gene sequence (T01-1) is closely related to the known isolate GS^19^, also from peach in China (Fig. 6B). Four homologs of the heat shock protein 70 (HSP70h) gene sequences from PBNSPaV isolates from samples T01 and T02 are also closely related to the known isolates Plm-WH-3 and WH-1, and three T01 HSP70h gene sequences are also closely related to the known isolates GS (Fig. 6B). Seven CP sequences from the ACLSV isolates from samples T01, T02, and T04 in our study all grouped together with the known isolates Z1 and Z3^23^ from peach trees in China (Fig. 6C). Six CP sequences from the APV1 isolates from T01 and T02 are closely related to each other and to the known isolates D2363 and D2367 (Fig. 6D). The three CP sequences from APV2 isolates from sample T04 are closely related to the APV2 isolate Bonsai^12^ from Japan (Fig. 7E). The three CGRMV CP sequences from isolate T02 are closely related to isolate F9^24^ from China (Fig. 7F). The three CP sequences from CNRMV isolates from sample T02 are closely related to isolates Pe-WH-18^25^ and 103-13 from China (Fig. 7G). For the few available sequences in GenBank for PaLV and the newly reported PeVD sequences, we analyzed their phylogenetic relationships using an outgroup. As expected, six RdRp and three CP sequences from PaLV and five PeVD replicase polyprotein gene sequences from isolates T01 and T02 were found to be closely related to the isolates in GenBank (Fig. 7H,I).

Sequence comparisons using the T01-T05 datasets showed that the NSPaV and PBNSPaV sequences in this study are highly variable, with the nucleotide sequence identities among the NSPaV RdRp gene sequences ranging from 89.4 to 99.6%, with comparable values of 93.2 to 99.5% for the PBNSPaV CP gene sequences. This contrasts with other gene sequences from ACLSV, APV1, APV2, CGRMV, CNRMV, PaLV, and PeVD that are highly homologous (96.7 to 100%) in identified isolates.

## Discussion

NGS technologies provide a powerful way to detect and identify viral pathogens with no prior knowledge of virus genome sequences^3,26^. This technology is finding increased applications in revealing the viromes that contribute to host phenotype and also the recent evolutionary history of RNA viruses^14–18,27,28^.

In this work, we studied co-infecting virus and viroid communities using NGS technology in five individual trees of two nectarine cultivars associated with different disease phenotypes, and then confirmed the identified viruses/viroids using RT-PCR in 36 samples from four cultivars. Identifying comparable plant materials with the same genetic background and cultivation history associated with the different disease symptoms can be difficult. In this study, some valuable samples were collected from trees in two small greenhouses that were grown under the same environmental conditions, with the same cultivation methods, pesticides, and fertilizers, which allowed for a direct comparison of the effects of co-infecting virus and viroid communities in trees with different disease phenotypes. The results indicated that the viral species present in viral communities isolated from the four different cultivars were diverse. A single tree, T02 of cultivar ‘Youtao 1233’ that showed symptoms of fruit pitting, harbored most of the nine different viruses/viroids from five families including PLMVd, NSPaV, PaLV, PBNSPaV, ACLSV, APV1, CGRMV, CNRMV, and PeVD, while T01, that did not show fruit pitting, harbored seven viruses/viroids from the above group in addition to CGRMV and CNRMV. To our knowledge, this is the first report that most identified viral species form co-infections in a individual peach tree, and also the first report of PeVD in peach in China. These results confirmed and extended the utilization of NGS to detect fruit tree viruses and to provide further insight into the complex multiple infections in individual trees and between different cultivars.

Combined genetic variation and sRNA data analyses of the co-infecting viruses/viroids in this study implied that viral synergisms and divergent sequence variants play an important role in determining disease symptoms. This includes synergisms among a few genera in the viral families *Betaflexiviridae*, *Closteroviridae*, and *Luteoviridae* and the possible effects on facilitating divergent virus sequence variants in nectarine symptom expression, increasing the titers of pathogenic viral genotypes.

Two functional classes of viral genes, a transcription-related RdRp gene and a structural CP gene, are often used for phylogenetic classification of plant viruses^19,23,29,30^. The HSP70h genes are used for phylogenetic classification of plant viruses in the family *Closteroviridae*^31^. A study by Qu *et al*. also showed that the evolutionary relationships of global PBNSPaV isolates can be reliably inferred using HSP70h sequences^19^.

Phylogenetic analyses of the identified 10 viruses and one viroid in the five individual nectarine trees using whole genome sequences, nearly complete genome sequences, and CP, RdRp, and HSP70h gene sequences showed that PLMVd, NSPaV, and PBNSPaV gene sequences were more divergent, and presented different sequence variants or recombinants. However, gene sequences from ACLSV, APV1, APV2, CGRMV, CNRMV, PaLV, and PeVD from the five individual trees showed much less variability. It should be noted that full genome sequences of the identified viruses could not be obtained in this study; however, the phylogenetic relationships were analyzed using two functional classes of viral genes that might be involved in viral synergisms.

Stem pitting has been diagnosed in peaches infected by tomato ringspot virus (ToRSV)^32^ and PBNSPaV^19^, and in nectarine infected by NSPaV^5^. The PBNSPaV peach isolate WH-1 causes trunk gummosis, cracking, necrosis, and stem pitting symptoms^19^. Based on the results of our phylogenetic analysis, CGRMV and CNRMV are most similar to apple stem pitting virus^24^, but the effects on fruit pitting in *Prunus* have not been reported.

Compared with sample T01 (no fruit pitting), CGRMV and CNRMV were only detected in the fruit pitting sample T02, and their CP sequences clustered into a single group with known peach isolates from China. Also, for PBNSPaV, the number of PBNSPaV-specific reads in T02 was higher than in T01, and the number of contigs in T02 that mapped to the known PBNSPaV isolate WH-1 was significantly higher. The NSPaV isolates in T02 and T01 were clustered into different groups, confirming that they represent divergent sequence variants. Taken together, this result indicates that the fruit pitting observed in the T02 nectarine tree could be the result of interactions between PBNSPaV in family *Closteroviridae*, CGRMV and CNRMV in family *Betaflexiviridae*, and NSPaV in family *Luteoviridae*. Therefore, we can speculate that CGRMVand CNRMV might possibly serve as “helper” viruses in the involvement of specific variants of either NSPaV or PBNSPaV or both in the expression of fruit pitting symptoms. Synergisms have been reported to occur between the members of the genera *Closteroviridae* and *Betaflexiviridae*, and the p10 silencing suppressor from grapevine virus A in the family *Betaflexiviridae* enhances the infectivity of a *Closterovirus*, beet yellows virus^33^, but the synergisms of serveral viral genera reported in this study is first discovered.

Again, samples T03, T04, and T05 showed different disease phenotypes; we detected PLMVd and NSPaV in the asymptomatic tree T03, PLMVd, NSPaV, APV2, and ACLSV in tree T04 with dimpled fruits, and only PLMVd in tree T05 with mottled leaves. In addition, of the five sampled nectarine trees, APV2 was only found in sample T04. This result was also confirmed in an additional five sampled trees with dimpled fruits using an RT-PCR screen. These results suggest that fruit dimpling at least may be related to the increase in APV2 and ACLSV titers. Previous studies have shown that, based on the amino acid sequences of the CP, ACLSV isolates have been classified into the types Z1/Z3 and Ta Tao5 from peach samples^23^, and P205 and B6 from apple samples^30^. In our study, seven ACLSV CP sequences from T01, T02, T03, and T04 were all closely related to isolate Z1. This result would seem to exclude a contribution of ACLSV to the dimpled fruit symptoms in T04. According to a previous report, APV2 infection could contribute to leaf symptoms in the GF305 peach indicator^12^, but we have not found documented fruit dimpling symptoms. We have stated that NSPaV sequences in this study are more divergent, and that the T04-1 sequence, one of three RdRp gene sequences for NSPaV, was found to be a recombinant. Taken togather, we infer that dimpled fruit symptoms also result from the synergistic effects of co-infection with NSPaV and APV2. As previously reported, some luteoviruses, such as groundnut rosette assistor virus^34^, serve as “helper” viruses for the transmission of other viruses that cause disease.

PLMVd is the only viroid shared by all five nectarine trees that displayed three leaf disease phenotypes (asymptomatic, bleached, and chlorotic mottle) in this study. Some studies have shown that PLMVd infection can be associated with albinism (peach calico, PC) and green mosaic symptoms, and revealed a close association between the albino phenotype and variants containing an insertion of 12–14 nt, folding into a hairpin capped by a U-rich loop in the proposed PLMVd branched secondary structure^21,35^. It is worth noting that we did not find this 12–14 nt insertion in this study. However, evolutionary analysis of PLMVd shows that the PLMVd sequences associated with symptomatic and asymptomatic trees do not all cluster together, and that there are intra-isolate divergent sequence variants present in every individual symptomatic tree. It has been suggested that divergent sequence variants of PLMVd co-infecting a single tree could contribute to host phenotype.

Biological data on the direct association of disease symptoms with the viruses identified in this study in fruit tree are scant in the literature. The main reasons for this are the fact that alternative herbaceous hosts are difficult to identify, and viral particles are very difficult to obtain in purified form for fruit tree viruses. Mixed viral communities that co-infect individual trees could increase viral genotypic complexity with implications for consequences to host pathology. However, increased application of NGS technology has resulted in the recent discovery of a large number of co-infecting viral communities in plants. Typically, this technology is used to identify candidate pathogens that may be associated with disease symptoms in plants^36^. In the present study, several valuable field samples with disease phenotype differences combined with NGS data and genetic analyses supported the association between multiple co-infecting viruses and the disease symptoms observed. The data reported in this study will be important for further study of the biological and epidemiological features of virus/viroid interactions in plant hosts.

## Methods

### Plant sources

In the spring of 2014, several 10-year-old trees of nectarine cultivars ‘Youtao 1233’ and ‘Youtao 126’ growing in greenhouse #1 in Daliang, Liaoning Province were found to have bleached leaves and rusty stem spots or fruit-pitting symptoms. In the spring of 2017, some 5-year-old trees of the nectarine cultivar ‘Zhongyou 4’ from greenhouse #2 (also in Daliang) were found to have symptoms of fruit dimpling but no visible leaf or fruit symptoms, and another group of trees had leaves that showed symptoms of chlorotic mottling. Some asymptomatic samples from 2-year-old trees of nectarine cultivar ‘Chaoyue 1’ from neighboring greenhouse #3 (also in Daliang) were also collected. The area of the three greenhouses is approximately 0.5–1.0 Chinese mu (1 mu = 666.7 square meters). The thirty-six symptomless and symptomatic tissue samples from greenhouses #1, #2, and #3 were collected and stored at −80 °C prior to use in the experiments. Of the 36 samples, the several different classes of symptoms were observed simultaneously on trees in greenhouses #1 and #2, which share the same environmental conditions as well as cultivation methods, pesticides, and fertilizers, and this allowed us to further study the etiological agent(s) associated with the different disease symptoms. Thus, five tissue samples from five different trees (two ‘Youtao 1233’ from greenhouse #1 and three ‘Zhongyou 4’ from greenouse #2) with or without disease symptoms on leaves and fruits were screened for viruses by sequencing the small RNAs using NGS as shown in Table 1 and Fig. 1.

### Viral communities identified by NGS in the small RNA libraries

Total RNA was extracted from each sample, and the small-RNA libraries were constructed using the NEB Multiplex Small RNA Library Prep kit (NEB, USA) following the manufacturer’s recommendations. Unique index codes were added to attribute the individual sequence reads to each sample library. The libraries were size-selected in 6% polyacrylamide gels prior to sequencing on an Illumina HiSeq. 2500 SE50 instrument (Biomarker Technologies Co., Ltd), and paired-end reads were generated.

The raw read data in fastq format were initially processed using in-house perl scripts. In this step, clean reads were obtained by removing reads containing adapters, reads containing multiple Ns (unknown bases), and low quality reads from the raw data. The reads were trimmed and cleaned by removing sequences smaller than 18 nt or longer than 35 nt. The phred quality scores (Q20 and Q30), GC-content, and sequence duplication level were calculated for the clean data. All downstream analyses were conducted with high quality clean data.

The sequence reads were assembled *de novo* into contigs using Velvet Software with k-mer = 17^37,38^. The contigs obtained were subsequently annotated by BlastN and BlastX searches of the Genbank virus and viroid Reference Sequence Database. sRNA reads that mapped to individual viral genomes were also tabulated to identify candidate viruses present in the analyzed nectarine samples.

### Virus and viroid detection with RT-PCR

Total nucleic acids were extracted from each sample using the RNAprep Pure Plant Kit (Tiangen Biotech (Beijing) Co., Ltd). Seventeen specific primer pairs (Table S2) were designed to amplify genomic regions corresponding to the CP, RdRp, or HSP70h genes from APV1, APV2, APV3, ACLSV, CGRMV, CNRMV, PBNSPaV, PeVD, NSPaV, PaLV, and GRGV, and also the complete genome sequence of PLMVd. The specific primer pairs to amplify nearly the complete genome of NSPaV are listed in Table S3. Reverse transcription (RT) was performed at 42 °C for 1 h using 1 μL of total RNA and 1 μL of oligo (dT) primer and 6-mer random primers in a 10 μL reaction volume containing Maloney murine leukemia virus (M-MLV) reverse transcriptase (Promega, Madison, WI, USA), according to the manufacturer′s protocol. Following RT, PCR assays were performed in 25 μL reaction volumes containing 1.5 μL of the RT reaction, 12.5 μL of 2X Taq Mix [Tiangen Biotech (Beijing) Co., Ltd.], 9.0 μL distilled water, and 1.0 μL (10 pmol) of the forward and reverse primers. The thermocycling conditions were as follows: an initial denaturation step of 5 min at 94 °C, followed by 35 cycles of 30 s at 94 °C, 30 s at 52 °C–55 °C, and 90 s at 72 °C, with a final extension step of 10 min at 72 °C.

RT-PCR products were purified using a PCR purification kit (AXygen), and the resulting DNA fragments were then cloned into the pMD18-T vector (Takara) for sequencing by the Sanger sequencing method. At least three clones of each amplified fragment were sequenced. Sequence reads were assembled using DNAMAN 6.0 (Lynnon Biosoft, Quebec, Canada).

### Phylogenetic analyses of the identified viruses/viroids

We amplified complete genome sequences for PLMVd, nearly complete genome sequences for NSPaV, and full-length or partial sequences of the CP, RdRp, and HSP70h genes for the identified viruses from the five tree samples T01, T02, T03, T04, and T05 using RT-PCR with specific primers (Tables S2 and S3). The PCR products were cloned and sequenced, with at least three clones sequenced from each sample tree. All CP, RdRp, and HSP70h gene sequences from the identified viruses were aligned and the flanking sequences in the amplified fragments were removed to obtain the full or partial-length gene sequences for each virus. In total, 17 PLMVd genomes (336 to 338 bp), one nearly complete NSPaV genome (4,578 bp), 11 NSPaV RdRp genes (987 bp), seven ACLSV CP genes (582 bp), six PBNSPaV partial CP genes (963 bp) and six partial HSP70h genes (587 bp), six APV1 CP genes (1,206 bp), three APV2 partial CP genes (1,182 bp), six PaLV partial RdRp genes (981 bp) and three CP genes (647 bp), five PeVD genes (695 bp), three CGRMV CP genes (807 bp), and three CNRMV CP genes (804 bp) were used in the phylogenetic analyses. We download other known complete genome, CP, and RdRp gene sequences from GenBank (www.ncbi.nlm.nih.gov) to determine the phylogenetic relationships with known viruses/viroids. If there are too many virus/viroid sequences deposited in GenBank except for recently-identified PeVD, PaLV, and NSPaV sequences, after filtering partial sequences, we only retrieved complete genome sequences homologous to each virus and a few representative sequences (with different disease symptoms) from the NCBI nucleotide database to use in phylogenetic tree construction. We aligned the genome or gene sequences using the ClustalW multiple alignment program and calculated a sequence identity matrix using BioEdit^39^ with the default parameters. The aligned sequences were checked for potential recombination events using RDP^13,40^. After sequence alignment, a phylogenetic tree was constructed using the maximum-likelihood (ML) method as implemented in MEGA6^41^, with confidence levels for the phylogroups estimated using 1,000 bootstrap replicates. The evolutionary history was inferred using the ML method based on the GTR + G model^13,42^.

## Supplementary information

Supplementary Tables

## Data Availability

The sequences from the virus/viriod isolates using in the phylogenetic analyses in the present work have been deposited with NCBI (https://www.ncbi.nlm.nih.gov) under the following accession numbers: 17 PLMVd genomes: MH974826-MH974842, 1 NSPaV nearly complete genome: MK361454, 11 NSPaV partial RdRp gene sequences: MK361443-361453, 6 PBNSPaV partial CP gene sequences: MK361484-361489, 7 PBNSPaV partial HSP70h gene sequences: MK361490-361496, 7 ACLSV CP genes: MK361436-361442, 6 APV1 CP genes: MK361455-361460, 3 APV2 partial CP gene sequences: MK361461-361463, 3 CGRMV CP genes: MK361464-361466, 3 CNRMV CP genes: MK361467-361469, 3 PaLV partial CP gene sequences: MK361470-361472, 6 PaLV partial RdRp gene sequences: MK361478-361483, 5 PeVD partial Replicase polyprotein gene sequences: MK361473-361477.
