# Supplementary material for: Analyses of virus/viroid communities in nectarine trees by next-generation sequencing and insight into viral synergisms implication in host disease symptoms
Source: Sci Rep. 2019 Aug 22;9:12261. doi: 10.1038/s41598-019-48714-z (PMC6706421; doi:10.1038/s41598-019-48714-z)
Supplement: Supplementary file 1 — Supplementary Tables [file 41598_2019_48714_MOESM1_ESM.pdf]

Supplementary Information

Analyses of virus/viroid communities in nectarine trees by next-generation sequencing and insight into viral synergisms implication in host disease symptoms

Yunxiao Xu[a], Shifang Li[a,b], Chengyong Na[c], Lijuan Yang[a], Meiguang Lu[a*]

[a] State Key Laboratory for Biology of Plant Diseases and Insect Pests, Institute of Plant Protection, Chinese Academy of Agricultural Sciences, Beijing, China;

[b] Chinese Academy of Tropical Agricultural Sciences, Hainan, China;

[c] The Agricultural Development and Service Center of WFD, Liaoning, China.

* Address correspondence to Meiguang Lu, mglu@ippcaas.cn

Supplementary Data

Supplementary Tables

Table S1 sRNA sequencing, contig assembly, and virus/viroid genome identification.

Table S2 Oligonucleotide primers used for amplification of virus and viroid sequences in this study.

Table S3 Oligonucleotide primers used for amplification of nearly complete NSPaV genome sequences in this study.

Table S4 Infection by individual viruses and viroids was verified in 36 nectarine tree samples by RT-PCR assays.

Table S1. sRNA sequencing, contig assembly, and virus/viroid genome identification.

**Sample**	**Number of raw sRNA reads**	**Clean sRNA reads**	**Mapped sRNA reads**	**Mapped sRNA/ Clean reads**	**Number of contigs**	**Contig length (nt)**
T01	22434184	20906239	1156297	5.53%	687	33-366
T02	23559998	22191401	2129850	9.60%	744	33-338
T03	30438485	28435618	316928	1.11%	52	33-474
T04	29588745	24849851	672912	2.71%	243	33-408
T05	27056398	25000656	566883	2.27%	15	33-84

Table S2 Oligonucleotide primers used for amplification of virus/viroid sequences.

Primer		Primer sequence (5′-3′)	Position [a]	Product size (bp)	References[b]
APV1	F	ATGAGTACCTCTTCTGCAACAG	CP	1210	This study
	R	ACCCTCAATCCATCTCCTTCC			
APV2	F	AGGTCCAGTGTGTGTAGATTGA	CP	1235	This study
	R	CAACAACAGTCTTGACCGGG			
APV3	F1	ACTGGTGAGTCTGTGGTAGT	CP	1389	This study
	R1	TGGGCGACTAATCCATCTCC			
	F2	ACTCTACAGGTACATTCGGAC	Replicase	489	Marais et al. 2016
	R2	CCATCAAGAACATGGTGCAGA			
ACLSV	F	TCTGCAAGAGAATTTCAGTT	CP	800	Lu et al. 2015
	R	GTCTACAGGCTATTTATTATAAG			
CGRMV	F	AGAGGGCATTGGTTACTGGT	CP	988	This study
	R	AAACGGGCACTCTGGTAAGA			
CNRMV	F	CTTCCAAGCAAGATTGTCGC	CP	1172	This study
	R	TTCGCCCCGTGTTGTAAGAC			
GRGV	F1	CACCCCTTCTCCCCATGTGAC	CP	441	Fan et al. 2016
	R1	ACAGCTGGGGCTTCAGGGTT			
	F2	TCCGCCCCGAGAAGCACTTTG	RdRp	233	Fan et al. 2016
	R2	AGAAGCTCGGAGAGGAGAGGG			
PBNSPaV	F	AGACAATCGATGCCTGCAAC	CP	1046	This study
	R	AGCCCTTCTAACAGTCACGT			
PeVD	F	CCGACTCTGTCATTGACTACCC	Replicase Polyprotein	695	This study
	R	GAGGACTCGTGCGGACTTTA			
NSPaV	F1	CACTGTGATCCCATCCCCTT	RdRp	1036	This study
	R1	AAACCAGAGTCCGATCCGAG			
	F2	AACATGTCGAGGTGGTGGAT	RdRp P1-P2 fusion	712	Lu et al. 2017
	R2	GCCCACATCTTCGACGATTC			
	F3	CCTGTACAGCCGAAGAAAGC	CP	474	Lu et al., 2017
	R3	ATGAGTGTGCAGGGTGATGA			

PaLV	F1	ATCCAGCATGATTCGTTCGT	RdRp	981	This study
	R1	CGTGACAGAGCCCATCTAAAA			
	F2	ACGATAACAATTCACCGCGC	CP	647	This study
	R2	GAGGAGCTGCGTCTACCTAG			
PLMVd	F	GGAATTCCTGTGATCCAGGTACCGCCGTAGAAACT	Genome	337	Ambros et al. 1998
	R	CTGGATCACACCCCCCTCGGAACCAACCGCT			

[a] CP, Coat protein; RdRp, RNA-dependent RNA polymerase.

[b]References:

1. Marais A, Faur, C, Candresse T. 2016. New Insights into Asian Prunus Viruses in the Light of NGS-Based Full Genome Sequencing. PLoS One. 11: e0146420.
2. Lu M, Wu B, Gao R, Zhang Z, Xiao H, Chen R, Li S. 2015. Preliminary investigation on cherry virus diseases and detection the pathogens in some regions of China. Plant Protection. 41: 98–103. (in Chinese).
3. Fan X, Dong Y, Zhang Z, Ren F, Hu G, Li Z, Zhou J. 2016. First Report of Grapevine red globe virus (GRGV) in Grapevines in China. Plant Dis. 100(11): 2340.
4. Lu M, Zhang C, Zhang Z, Wang C, Li S. 2017. Nectarine stem-pitting-associated virus detected in peach trees in China. Plant Dis. 101(3): 513-513.
5. Ambros S, Hernandez C, Desvignes JC, Flores R. 1998. Genomic Structure of Three Phenotypically Different Isolates of Peach Latent Mosaic Viroid: Implications of the Existence of Constraints Limiting the Heterogeneity of Viroid Quasispecies. J Virol. 72(9): 7397–7406.

Table S3 Oligonucleotide primers used for amplification of NSPaV genomic sequences.

Primer		Primer sequence (5′-3′)	Position	Product size (bp)
NSPaV-P1	F	CACTGTGATCCCATCCCCTT	108-127	1036
	R	AAACCAGAGTCCGATCCGAG	1143-1124	
NSPaV-P2	F	ATGACACGCTGGATGAC	529-545	2422
	R	GCTGTAGCTGCTTGCTG	2950-2934	
NSPaV-P3	F	AAGCGGCAAAGAAGAAATCC	2882-2901	1338
	R	TAGTCGCAACTGACCGTGGG	4219-4200	
NSPaV-P4	F	ATCTGGGAATTACAACGGACAT	3672-3693	1014
	R	CAACGTGGACCTTTGTTTGC	4685-4666	

Table S4 Infection by individual viruses and viroids was verified in 36 nectarine samples by RT-PCR.

Samples	Collecting Greenhous	Cultivar	Tree age	Leaf symptoms	Fruit symptoms	RT-PCR results											
						APV1	APV2	APV3	ACLSV	CGRMV	CNRMV	GRGV	PBNSPaV	NSPaV	PaLV	PeVD	PLMVd
P1	#3	Chaoyue 1	2	Asymptomatic	Asymptomatic	-	-	-	-	-	-	-	-	-	-	-	+
P2	#3	Chaoyue 1	2	Asymptomatic	Asymptomatic	-	-	-	-	-	-	-	-	-	-	-	+
P3	#3	Chaoyue 1	2	Asymptomatic	Asymptomatic	-	-	-	-	-	-	-	-	-	-	-	+
P12	#2	Zhongyou 4	5	Chlorotic mottling	Asymptomatic	-	-	-	-	-	-	-	-	-	-	-	+
P13	#2	Zhongyou 4	5	Chlorotic mottling	Asymptomatic	-	-	-	-	-	-	-	-	-	-	-	+
P14(T05)	#2	Zhongyou 4	5	Chlorotic mottling	Asymptomatic	-	-	-	-	-	-	-	-	-	-	-	+
P15	#2	Zhongyou 4	5	Chlorotic mottling	Asymptomatic	-	-	-	-	-	-	-	+	-	-	-	+
P16	#2	Zhongyou 4	5	Asymptomatic	unknown	-	-	-	-	-	-	-	-	+	-	-	+
P17	#2	Zhongyou 4	5	Asymptomatic	unknown	-	-	-	-	-	-	-	-	+	-	-	+
P18	#2	Zhongyou 4	5	Asymptomatic	unknown	-	-	-	-	-	-	-	-	+	-	-	+
P19	#2	Zhongyou 4	5	Asymptomatic	unknown	-	-	-	-	-	-	-	-	+	-	-	+
P25(T03)	#2	Zhongyou 4	5	Asymptomatic	Asymptomatic	-	-	-	-	-	-	-	-	+	-	-	+
P25-F(S06)	#2	Zhongyou 4	5	Asymptomatic	Asymptomatic	-	-	-	-	-	-	-	-	+	-	-	+
P26	#2	Zhongyou 4	5	Asymptomatic	Asymptomatic	-	-	-	-	-	-	-	-	+	-	-	+
P26-F	#2	Zhongyou 4	5	Asymptomatic	Asymptomatic	-	-	-	-	-	-	-	-	+	-	-	+
P27	#2	Zhongyou 4	5	Asymptomatic	Asymptomatic	-	-	-	-	-	-	-	-	+	-	-	+
P20	#2	Zhongyou 4	5	Asymptomatic	dimpling	-	+	-	+	-	-	-	-	+	-	-	+
P20-F	#2	Zhongyou 4	5	Asymptomatic	dimpling	-	+	-	+	-	-	-	-	+	-	-	+
P21(T04)	#2	Zhongyou 4	5	Asymptomatic	dimpling	-	+	-	+	-	-	-	-	+	-	-	+
P21-F(S05)	#2	Zhongyou 4	5	Asymptomatic	dimpling	-	+	-	+	-	-	-	-	+	-	-	+

continue

Samples	Collecting Greenhous	Cultivar	Tree age	Leaf symptoms	Fruit symptoms	RT-PCR results											
						APV1	APV2	APV3	ACLSV	CGRMV	CNRMV	GRGV	PBNSPaV	NSPaV	PaLV	PeVD	PLMVd
P23	#2	Zhongyou 4	5	Asymptomatic	dimpling	-	+	-	+	-	-	-	-	+	-	-	+
P23-F	#2	Zhongyou 4	5	Asymptomatic	dimpling	-	+	-	+	-	-	-	-	+	-	-	+
P24	#2	Zhongyou 4	5	Asymptomatic	dimpling	-	+	-	+	-	-	-	-	+	-	-	+
P24-F	#2	Zhongyou 4	5	Asymptomatic	dimpling	-	+	-	+	-	-	-	-	+	-	-	+
N8(T01)	#1	Youtao 1233	10	Bleached	Asymptomatic	+	-	-	+	-	-	-	+	+	+	+	+
N8-2	#1	Youtao 1233	10	Bleached	Asymptomatic	+	-	-	+	-	-	-	+	+	+	+	+
N9(T02)	#1	Youtao 1233	10	Bleached	pitting	+	-	-	+	+	+	-	+	+	+	+	+
N9-2	#1	Youtao 1233	10	Bleached	pitting	+	-	-	+	+	+	-	+	+	+	+	+
N7-2	#1	Youtao 126	10	Bleached	unknown	-	-	-	-	-	-	-	-	-	+	-	+
N7(S09)	#1	Youtao 126	10	Bleached	unknown	-	-	-	-	-	-	-	-	-	+	-	+
Y3-1	#1	Youtao 126	10	Bleached	unknown	-	-	-	-	-	-	-	-	-	-	-	+
Y3-2	#1	Youtao 126	10	Symptomless leaf of bleached	unknown	-	-	-	-	-	-	-	-	-	-	-	+
Y4	#1	Youtao 126	10	Bleached	unknown	-	-	-	-	-	-	-	-	-	+	-	+
Y6	#1	Youtao 126	10	Bleached	unknown	-	-	-	-	-	-	-	-	-	-	-	+
Y7	#1	Youtao 126	10	Bleached	unknown	+	+	-	+	-	-	-	-	-	+	+	+
Y8	#1	Youtao 126	10	Bleached	unknown	-	-	-	-	-	-	-	-	-	-	-	+

Of the 36 samples, P20-F, P21-F, P23-F, P24-F, P25-F, and P26-F were from fruit tissue. P14(T03), P25(T04), P21(T05), N8(T01), N9(T02), N7(S09), P25-F(S06), and P21-F(S05) were also screened for viruses and viroids by sequencing the small RNAs using next generation sequencing (NGS). ‘+’, positive. ‘-’, negative.
